# Factors Influencing Contraceptive Use Among Sexually Active U.S. Middle and High School Students, 2015 to 2019

**DOI:** 10.7759/cureus.20803

**Published:** 2021-12-29

**Authors:** Valerie S Chuy, Geethanjali Rajagopal, Rachna Talluri, An-Lin Cheng, Lawrence Dall

**Affiliations:** 1 Obstetrics and Gynecology, University of Missouri Kansas City School of Medicine, Kansas City, USA; 2 Internal Medicine, University of Missouri Kansas City School of Medicine, Kansas City, USA; 3 Internal Medicine, University of Missouri Kansas City, Kansas City, USA; 4 Biomedical and Health Informatics, University of Missouri Kansas City School of Medicine, Kansas City, USA

**Keywords:** contraception, condom, adolescent, sexual intercourse, sexually transmitted diseases, pregnancy

## Abstract

Objective

The objective of this study is to see whether factors including the age of first sexual intercourse, sexual orientation, age at the time of survey administration, race/ethnicity, and sex affect condom and other types of contraceptive usage among United States middle and high school students.

Methods

We analyzed data from the Centers for Disease Control’s Youth Risk Behavior Surveys from the years 2015 to 2019. Students were asked questions about condom and specific type of contraceptive use (e.g. birth control pills, intrauterine device/implant, shot/patch/ring, withdrawal), age of first sexual intercourse, and sexual orientation, as well as demographic questions. Using a logistic regression model, we tested the linear effects on condom and contraception investigated variables.

Results

Differing sexual orientations had a significant relationship with both condom and contraceptive usage, with those who identified as lesbian or gay being least likely to use contraception as opposed to those who identified as straight, bisexual, or unsure. Female participants were 31.6% less likely to use contraception overall and 41.7% less likely to use condoms in comparison to male participants. With a one-year increase in the age of first sexual intercourse, contraceptive use overall increased by 23% and condom use specifically increased by 17%. With a one-year increase in a participant’s age at the time of survey administration, contraceptive use decreased by 7.4% and condom use decreased by 21%. Between the years 2015 to 2019, there was an average decrease of 5.3% in the usage of condoms.

Conclusion

We found significant differences in contraceptive and/or condom usage between students of different sexual orientations, sex, age of first sexual intercourse, age at the time of survey administration, and between different years studied. These differences could be attributed to differences in sex education, cultural background, and availability of resources. Further investigations should be conducted to delineate these differences.

## Introduction

Though the United States adolescent pregnancy rates have been declining, according to the American College of Obstetricians and Gynecologists (ACOG), the United States continues to have the “highest adolescent pregnancy and birth rates among developed countries” [1]. This disproportionately affects certain groups: Black and Hispanic adolescents of lower socioeconomic status. Furthermore, childbearing leads to continued adverse effects on socioeconomic status for the mother and her family.

Disparities regarding contraception availability in clinics and schools, as well as comprehensive sex education, have been cited as possible reasons for the decrease in adolescent pregnancy across the United States and different racial/ethnic groups. Programs addressing these disparities, such as school-based health centers, have not been applied to a great enough scale to notice if they significantly influence adolescent pregnancy rates [1].

Additionally, sexually transmitted disease rates have continued to climb, specifically among adolescents. In 2019, the highest rates of chlamydia cases happened in 15- to 19-year-old females. Similarly, reported gonorrhea rates were also higher in adolescent and young adult populations [2]. This higher prevalence of cases may reflect a difference in condom use between younger and older populations.

A study of women receiving abortion services in 2000-2001 showed that almost half did not use contraception. Participants who did not use contraception primarily cited perception of low pregnancy risk, whereas others listed concerns about contraception use and issues with access to contraception side effects and previous difficulties with contraception methods [3].

The need for increased contraceptive knowledge is demonstrated in long-standing gaps in young adults’ knowledge about contraceptive methods. In a study conducted at Guttmacher Institute, male and female participants aged 18-29 years were asked about their perceptions and knowledge regarding the use of contraception to see if there was a correlation with a woman’s risk of unintended pregnancy. The results from the study demonstrated that more than half of the men and a quarter of the women received low skills on contraceptive knowledge and “six in 10 underestimated the effectiveness of oral contraceptives” [4]. On the other hand, an increase in contraceptive knowledge led to decreased unprotected sexual intercourse within the following three months and an increased chance of using hormonal or long-acting reversible contraception. Research expanding contraceptive knowledge and usage to an adolescent population is limited.

The purpose of this study is to see whether factors including age of first sexual intercourse, sexual orientation, age at the time of survey administration, race/ethnicity, and sex affect condom and other types of contraceptive usage among United States middle and high school students. As contraceptive use and condom use are known methods to prevent pregnancy and sexually transmitted diseases respectively, it is valuable to identify factors that influence the use of these methods.

## Materials and methods

Our study was a retrospective analysis of the Centers for Disease Control and Prevention (CDC) provided data that was available to the public. The CDC’s Youth Risk Behavior Surveys (YRBS) is a national biennial paper survey that evaluates health risk behaviors among students in grades 6-12 at public and private schools across the United States. Students anonymously answered multiple-choice questions about their health risk behaviors. As per school regulations, parental permission was obtained. All items on the survey were deemed reliable [5]. The CDC received Institutional Review Board (IRB) approval for YRBS study design and data collection. Our study did not include any identifiable characteristics of individuals who participated in the study. Therefore, we did not require IRB review for this study.

We analyzed records from YRBS from the years 2015, 2017, and 2019. Surveys administered in these three years asked students “The last time you had sexual intercourse, did you or your partner use a condom?” Students were also asked, “The last time you had sexual intercourse, what one method did you or your partner use to prevent pregnancy?” Contraception is defined as “the intentional prevention of conception through the use of various devices, sexual practices, chemicals, drugs, or surgical procedures” [6]. Another question that students were asked was “How old were you when you had sexual intercourse for the first time?” The available answer choices for these questions are included in Table 1. Responses from students who answered “I have never had sexual intercourse” for either question were excluded from the study, as well as participants who did not answer all of the analyzed questions. These students were excluded as our study was focused on sexually active adolescents. Students were also asked demographic questions regarding age (at the time of survey administration), sex, race/ethnicity, and sexual orientation. Bar graphs were created to demonstrate the percentage of students who chose a given contraceptive method.

**Table 1:** YRBS Questions and Responses IUD, intrauterine device; YRBS, Youth Risk Behavior Surveys

Question	Response
The last time you had sexual intercourse, did you or your partner use a condom?	A. I have never had sexual intercourse. B. Yes C. No
The last time you had sexual intercourse, what one method did you or your partner use to prevent pregnancy? (Select only one response.)	A. I have never had sexual intercourse. B. No method was used to prevent pregnancy. C. Birth control pills. D. Condoms. E. An IUD (such as Mirena or ParaGard) or implant (such as Implanon or Nexplanon). F. A shot (such as Depo-Provera), patch (such as Ortho Evra), or birth control ring (such as NuvaRing). G. Withdrawal or some other method. H. Not sure
Which of the following best describes you?	A. Heterosexual (straight). B. Gay or lesbian. C. Bisexual. D. Not sure
How old were you when you had sexual intercourse for the first time?	A. I have never had sexual intercourse. B. 11 years old or younger. C. 12 years old. D. 13 years old. E. 14 years old. F. 15 years old. G. 16 years old. H. 17 years old or older

Then, a logistic regression model was used to estimate and test the linear effects on condom and contraception using factors such as the age of first sexual intercourse, year of study, race/ethnicity, sex, and sexual orientation while accounting for age at the time of study. Logistic regressions were conducted to examine the relationship between independent variables and outcome variables. We entered all interested variables in the model after making sure that there was no violation of multicollinearity (both models with variance inflation factor [VIF] all less than 2). We included variables that were not significant at the bivariate level because we wanted to control that bias in the final model. Chi-square tests were conducted to examine bivariate relationship between independent and dependent variables. T statistics were estimated and examined as part of logistic regression outcome. The level of significance is set at 0.05. Statistical analysis was performed utilizing SAS version 9.4 (SAS Institute Inc., Cary, NC). Odds ratio for the logistic regression model is shown in Table 2 and Table 3 for condom and contraception use, respectively.

**Table 2:** Odds Ratio Table for Factors Influencing Condom Use

Factor	Odds Ratio	95% Confidence Interval		p-Value
		Lower Limit	Upper Limit	
Sex	0.583	0.544	0.626	<0.0001
Year	0.947	0.908	0.987	0.0099
Age at the time of survey administration	0.790	0.764	0.816	<0.0001
Age at the time of first sexual intercourse	1.258	1.228	1.290	<0.0001
Sexual orientation: gay or lesbian vs straight	0.326	0.260	0.411	<0.0001
Sexual orientation: bisexual vs straight	0.790	0.702	0.888	<0.0001
Sexual orientation: not sure vs straight	0.828	0.668	1.026	<0.0001

**Table 3:** Odds Ratio Table for Factors Influencing Contraceptive Use

Factor	Odds Ratio	95% Confidence Interval		p-Value
		Lower Limit	Upper Limit	
Sex	0.684	0.617	0.758	<0.0001
Age at the time of survey administration	0.926	0.886	0.968	0.0007
Age at the time of first sexual intercourse	1.258	1.218	1.300	<0.0001
Sexual orientation: gay or lesbian vs straight	0.113	0.090	0.142	<0 .0001
Sexual orientation: bisexual vs straight	0.570	0.492	0.661	<0.0001
Sexual orientation: not sure vs straight	0.621	0.475	0.812	<0.0001

When analyzing contraception within the logistic regression model, contraception was defined as a binary variable. Contraceptive use responses were divided into “no contraception used” and “contraception used” with the first category including responses “no method used to prevent pregnancy” and “not sure” and the latter category including the rest of the six responses.

## Results

There were a total of 44,067 participants in the YRBS survey between the years 2015 and 2019. Studied population included 38,288 participants that answered yes to the questions of “Have you ever had sexual intercourse?” Participants that answered no to this question were excluded from the study. Of this population, 50.76% were females. Mean age of this population was 15.98 years. Regarding sexual orientation, 85.72% of the population identified as straight, 2.54% identified as gay or lesbian, and 7.68% identified as bisexual; 46.07% of participants identified as White.

**Figure 1:**
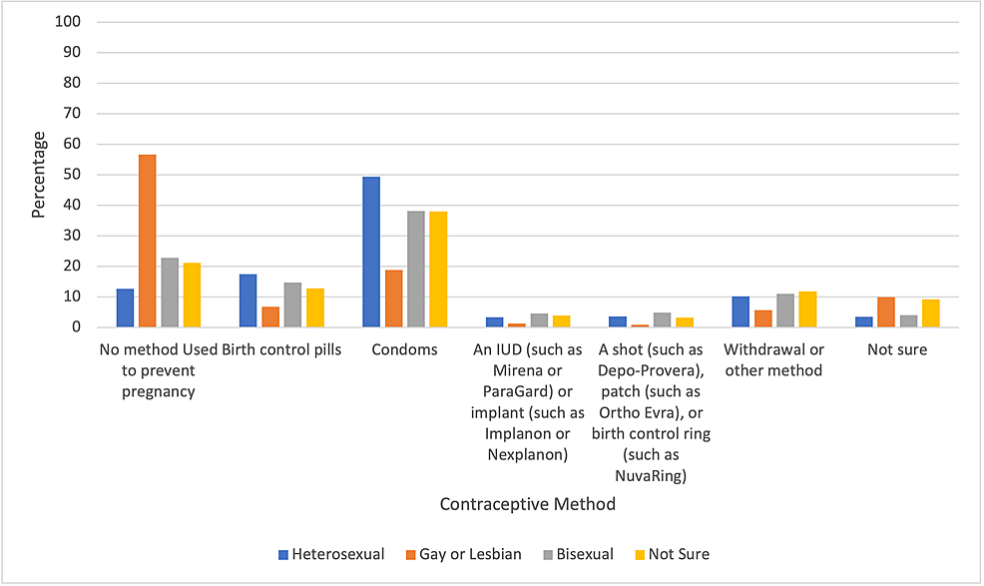
Percentages of Contraceptive Method Types by Sexual Orientation

There were statistically significant differences in contraceptive usage among adolescents that identified with different sexual orientations (Figure 1). Adolescents that identified as gay or lesbian were 88.7% less likely to use contraception than those who identified as heterosexual. In comparison to individuals who identified as heterosexual, those who identified as bisexual were 43% less likely to use contraception. Individuals that were not sure about their sexual identity were 37.9% less likely to use contraception than heterosexual individuals. There was also a statistically significant difference in condom usage between adolescents of different sexual orientations. Adolescents that identified as gay or lesbian were 67.4% less likely to use condoms than those who identified as heterosexual. Adolescents who identified as bisexual were 21% less likely to use condoms than those who identified as heterosexual. Adolescents who stated that they were not sure about their sexual identity were 17.2% less likely to use condoms than those who identified as heterosexual.

A statistically significant difference in contraceptive usage was found between male and female participants. Female participants were 31.6% less likely to use contraception than male participants. Females were 41.7% less likely to use condoms than male participants.

**Figure 2:**
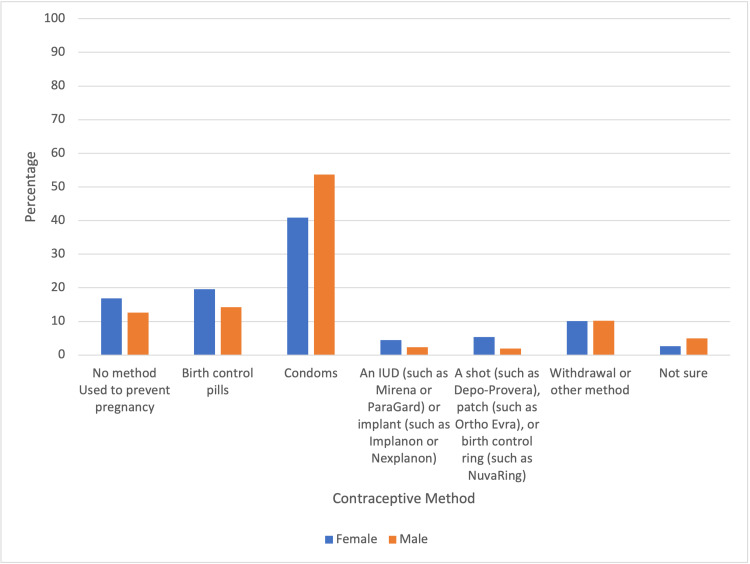
Percentages of Contraceptive Method Types by Gender

There was a statistically significant difference between usage of contraception among middle and high school students based on age of first sexual intercourse (Figure 2). With a one-year increase in the age at the time of first sexual intercourse, the chance of contraceptive usage is increased by 23%. Also, 11% of adolescents who had their first sexual intercourse at 11 years or younger reported usage of birth control in comparison to 17.5% of adolescents who were 17 years or older. There was a statistically significant difference between usage of condoms among middle and high school students based on age of first sexual intercourse. With a one-year increase in the age of first sexual intercourse, the chance of condom usage increased by 17%. In other words, delaying sexual intercourse by one year increases the chance of condom usage.

**Figure 3:**
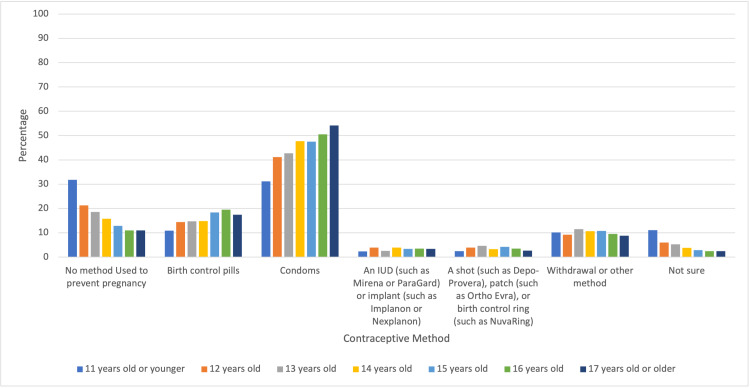
Percentages of Contraceptive Method Types by Age at the Time of First Sexual Intercourse

With a one-year increase in age at the time of survey administration, contraceptive use decreased by 7.4% and condom use decreased by 21% (Figure 3). While a one-year increase in age of first sexual intercourse corresponded to an increase in condom and contraceptive use, a one-year increase in age at the time of survey administration resulted in a decrease in condom and contraceptive use.

**Figure 4:**
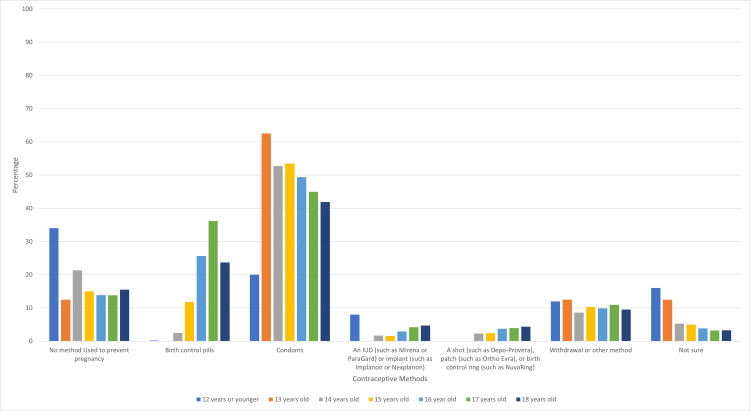
Percentage of Contraceptive Method Types by Age at the Time of Survey Administration

There was no statistically significant difference between usage of contraception or condoms among middle and high school students between the different races/ethnicities studied. However, the percentage of students who used no method to prevent pregnancy was highest among Black/African American students (20.1%) and Hispanic/Latino students (19.5%) and lowest among White students (10.8%) (Figure 4).

**Figure 5:**
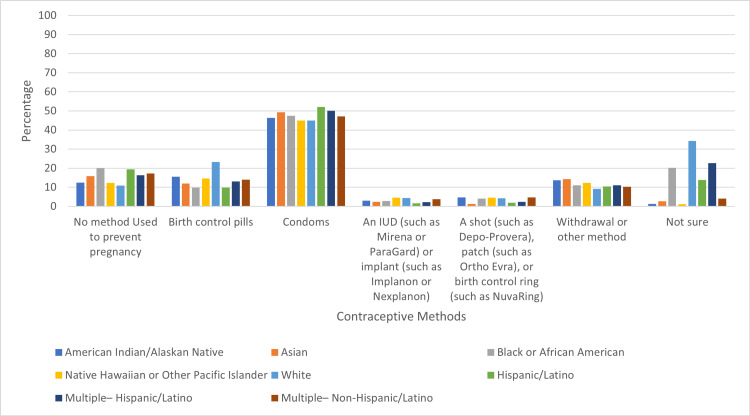
Percentage of Contraceptive Method Types by Race/Ethnicity

**Figure 6:**
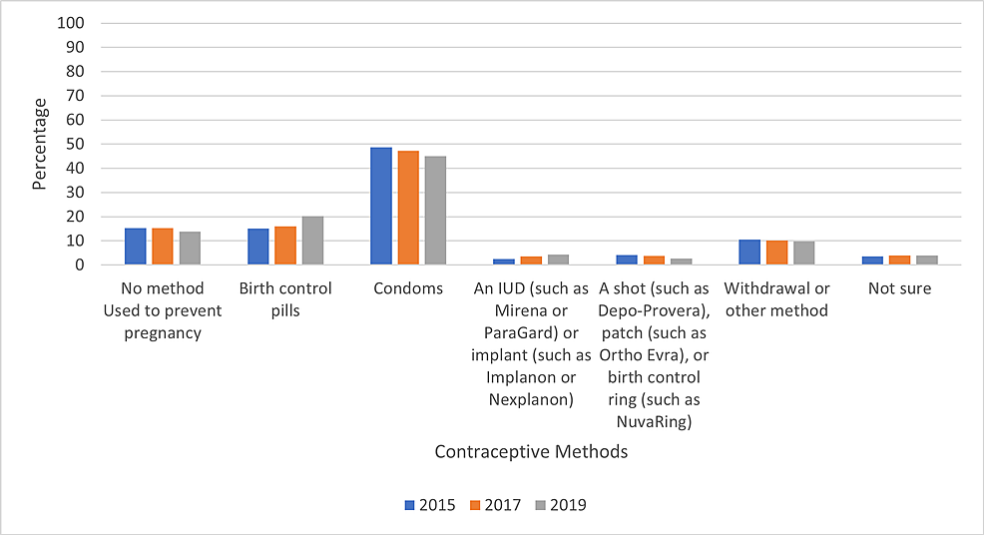
Percentage of Contraceptive Method Types by Year of Survey Administration

There was no statistically significant difference in contraceptive use in middle and high school students between the years 2015 to 2019, but there was a statistically significant decrease of 5.3% in usage of condoms among students between the years (Figures 5, 6).

## Discussion

Of the investigated variables, we found many statistically significant relationships that affected contraception and condom usage in middle and high school students in the United States.

Identifying with different sexual orientations had a statistically significant difference in both condom and contraceptive usage. Individuals that identified as lesbian or gay were least likely to use both condoms and contraception as opposed to those who identified as straight, bisexual, or unsure. These findings are consistent with previous studies of female populations that have shown decreased contraception usage among those that identify as bisexual or homosexual in comparison to those that identify as heterosexual [7-8]. In a previous study, focus groups of queer women have shown that this could possibly be due to multiple barriers to obtaining contraception such as perceived discrimination from health care professionals or feelings of being excluded from the conversation regarding contraception [9]. Comprehensive sexual education that is inclusive of varying sexual orientations could be valuable to increasing contraceptive use among those populations.

There appeared to be a statistically significant difference between condom and contraceptive usage among males and females, with males being more likely to use both condoms and contraception in general. This could likely be due to societal norms that often place the expectation of condom usage on males. For example, one study showed that women felt that men were likely to be critical of females that carried condoms as opposed to men [10]. Studies have also shown that women are less likely to use condoms if they felt that their partner was opposed to it, while men were less likely to be influenced by their perceived opinion of their partner’s beliefs [11].

One reason why contraception and condom usage increased with age of first intercourse could be due to an increase in exposure to sexual education if sexual activity is delayed later in adolescence. In a study conducted by John Hopkins University, sexual education and contraceptive use were studied from 2011 to 2017. Across all years studied, respondents who had sex education before their first sexual intercourse were less likely to have sex before age 15 [12]. In addition, the population of students was more likely to have used any or more effective contraceptive options such as birth control. Another reason could possibly be linked to greater sexual risk behavior among adolescents who have an earlier age of first sexual intercourse. In a study looking at the 2003-2015 National Youth Risk Behavior Surveys, variations in condom use were linked to a greater risk of sexually transmitted diseases in female students who reported higher risk sexual behaviors. These higher risk sexual behaviors included alcohol and illicit drug use before last sexual intercourse, four or more sex partners, two or more sex partners in the past 3 months, lack of condom use, and sexual intercourse before age 13 [13].

While there have been many previous studies looking at age at the time of first sexual intercourse, there have not been many studies analyzing effects of age on contraception and condom use. Our finding that condom and contraceptive use statistically decreased with increasing age at the time of survey administration is novel.

While our study showed no statistical difference in contraception use or condom use between the different races, our graphs did show a higher proportion of White students reporting using some form of contraception in comparison to other races. Black/African American students and Hispanic students had the lowest reported percentage of contraceptive usage. A June 2021 national study looked at racial/ethnic disparities in women receiving sexual health care/education and found that White women were more likely to receive access to birth control than Black or Hispanic women. The study, however, found that when adjusting for access to sexual health education, racial disparities were decreased [14].

There was no statistically significant change in overall contraceptive use between the years studied, but there was a statistically significant decrease in condom use between 2015 and 2019. The decrease in condom use in the four-year span of this study could possibly be attributed to the lack of strong sex education in some schools. A 2016 review article from the Journal of Adolescent Health notes that many schools promote “abstinence only until marriage” (AOUM) as part of school sex education despite lack of efficacy of AUOM education to reduce risky sexual behaviors in adolescents and improve reproductive health outcomes [15]. A 2021 report by the Guttmacher Institute shows that while 39 states and District of Columbia (DC) mandate sex education/HIV education, only 20 states and DC require education to include information on contraception. The same report found that 39 states require providing information on abstinence and 19 states require “education on importance of engaging in sexual activity only within marriage” [16]. To increase rates of contraception/condom use, one suggestion could be to have schools implement formal education on safe sex practices including types of contraception available, areas to get contraception, and discussion on safe sex practices with partners.

Some limitations of this study should be considered. The study only examined students enrolled in public or private schools across the United States. It does not examine the safe sex practices of adolescents who are homeschooled or who do not attend school. Students were only given the ability to pick one answer choice for type of contraceptive use, making it difficult to assess whether students were using multiple forms of contraception. Additionally, if adolescents used a different contraceptive method in the past, it is unclear whether they are choosing their current or past form of contraception. We were also unable to assess methods used to primarily prevent sexually transmitted diseases, outside of condoms. The study included both males and females who answered questions regarding contraceptive methods. As much of the contraceptive methods listed in the question (intrauterine device [IUD], implant, oral contraceptive pill [OCP]) rely on female participants, males might not have full knowledge of the contraceptive method used. Though student participants were aware that the survey was anonymous, some students could have neglected to truthfully answer questions regarding safe sex practices in fear of the consequences of answering truthfully.

Further investigations of this study could include examining whether schools that have standardized education on safe sex practices (including promoting use of condoms and contraceptives) influence adolescent use of condoms and other types of contraception. Analyzing this data could help solidify a possible need for more health education in schools. Concurrently, analyzing the regions in the United States where contraceptive usage or education on safe sex practices is low could help educators determine where incorporation of health education in school curricula is most important. Students could be asked whether they have access to certain methods of contraception and their barriers to obtaining said contraception. These data could help elucidate whether limited use of certain contraceptive methods is due to difficulty obtaining contraception or other causes. Methods such as IUDs, implants, and even OCPs are often very expensive without insurance. Students could also be interviewed about their personal decisions on using contraception or lack thereof. As a result, investigators can gain insight into whether contraceptive usage is most influenced by school safe sex education, availability of contraceptive methods, or familial and cultural perceptions on safe sex practices.

## Conclusions

When compared to other developed countries, the United States has one of the highest rates of adolescent pregnancy. In addition, rates of adolescent sexually transmitted diseases have been increasing in recent years. This could be attributed to poor condom and contraceptive usage among adolescents. We analyzed CDC’s YRBS questionnaires from the years 2015 to 2019 to investigate trends in contraceptive and condom usage among sexually active middle and high school students across the United States. We found statistically significant differences in contraceptive and/or condom usage between students of different sexual orientation, sex, age of first intercourse, age at the time of survey administration, and between the different years studied. These differences could be attributed to sex education, cultural background, and availability of resources. Contraceptive use could be improved among different populations analyzed through comprehensive sexual education and removal of barriers to contraception. Further investigations should be conducted to delineate these differences.
